# Three Steps Hybrid Treatment: En Bloc Resection, Endovascular Treatment and Microsurgical Reconstruction. Case Report of a Giant Dorsum‐Abdominal‐Pelvic Arteriovenous Malformation

**DOI:** 10.1002/micr.70194

**Published:** 2026-02-20

**Authors:** Iulia Elena Marin, Sara Tamburello, Francesco Mori, Alice Letizia Andreoli, Innocenti Alessandro, Raffaella Santi, Giulio Menichini

**Affiliations:** ^1^ Department of Plastic Reconstructive and Microsurgery AOU Careggi Florence Italy; ^2^ IRCCS Istituto Ortopedico Rizzoli Bologna Italy; ^3^ Pathology Section, Department of Health Sciences University of Florence Florence Italy

**Keywords:** DIEP flap, giant AVM, hybrid treatment, microsurgery, plastic reconstructive surgery

## Abstract

Arteriovenous malformations (AVMs) are a rare condition involving the trunk. We present a case of a 55‐year‐old woman affected by a giant dorsum‐abdominal‐pelvic AVM, even after multiple endovascular and surgical treatments over decades which required a different surgical approach. AngioCT scan was performed to identify the feeding vessels, the mass' extension and involvement of underlying tissues. Multidisciplinary discussion agreed on multistep treatment. First, we performed an en bloc resection starting from the right pelvis, moving cranially and laterally toward the right armpit, then removing medially toward the vertebral column, leaving a defect of 30 × 10 cm. Then a selective angiography and a paravertebral embolization of remaining AVM at the T7‐level were performed. Reconstruction was then completed with a left hemiabdomen DIEP free flap. We anastomosed one branch of the DIEA to the SCIA and one comitantae vein to the SCIV to assure lateral flow. Then we performed the anastomosis between the DIEA and the second comitantae vein respectively to a branch of TDA and to a branch of the serratus vein. The follow up at 6 months has satisfactory results for both patient and surgeon. This challenging case could provide an alternative for radical surgical excision combined with an endovascular control and microsurgical reconstruction. We describe what we believe to be the first reported giant AVM treated with this sequence multistep surgeries.

## 1 Introduction

Arteriovenous malformations (AVMs) are characterized by arteriovenous shunts, in which one or multiple arterial pedicles feed into a vascular nidus, and by the absence of capillaries between the two of them. This particular anatomy leads to a very fragile structure. It can easily rupture, causing excessive and uncontrolled bleeding, as well as causing an alteration of the gas homeostasis around it, since it does not possess capillaries and so it cannot assure a normal exchange of oxygen and metabolites (Young and Mulliken 1988). These malformations are more commonly linked to the central nervous system, and they may cause symptoms even in very small dimensions; major dimensions are characterized by a quite high mortality and an even higher morbidity. This has lead the research attention to intra‐cerebral AVMs, but less has been researched, explained, and treated about the extra‐cerebral AVMs. The common clinical manifestations of AVMs of the trunk and extremities are a pulsating mass, pain, ulceration, bleeding, tissue necrosis, enlargement of draining vein, and venous hypertension and/or cardiac failure (Gomes and Bernatz 1970). The Schobinger classification in IV stages for AVMs is established and valuable for the clinical assessment of the actual condition of the vascular anomaly and risk stratification for potential indications to treat (Kohout et al. 1998; Liu et al. 2010). An inappropriate treatment strategy (e.g., partial excision, ligation, or endovascular occlusion of the feeding artery) only stimulates the AVM lesion into a proliferative state, resulting in aggressive growth with uncontrollable complications (Liu et al. 2010). This is what happened in the case of this 55‐year‐old woman we are about to discuss. We report here our combined multistep management of a recurrent major AVM with an extension from the dorsum to the pelvis of the right hemisoma. The surgical resection of an AVM lesion carries the risks of extensive intraoperative hemorrhage, incomplete removal of the AVM nidus, surrounding organ or tissue injuries, and high recurrence rates (Kohout et al. 1998; Visser et al. 2011; Spiotta et al. 2011). Therefore, the combination of surgery with endovascular treatment with embolic agents at specific time set points demonstrated to be crucial in the radical treatment of our case. We would like to share our treatment plan with the scientific community for good clinical practice purposes taking into account the rarity and complexity of this case.

## 2 Case Report

We present a case of a 55‐year‐old woman with a giant AVM involving her pelvis, abdomen, and dorsum. The patient complained of continuous subtle pain exacerbated with compression and activity. During anamnesis, a mole‐like lesion involving the right hemithorax since birth was reported. At the age of 20, due to the growing dimensions and pain, the first surgery was performed, where a partial resection was completed in another center. After 4 years, due to the expedited growth, the lesion was treated by percutaneous vascular surgery combined with another partial surgical removal. At the age of 28, the patient started to be treated endovascularly with attempts at embolization of what developed as an increasing mass. Since 1997–2019 spiral embolization, ultrasound‐guided alcohol embolization, and sclerotherapy embolization for a minimum of 15 percutaneous procedures and also 2 cycles of laser therapy were performed with just little success. The treatment involved many hospitals in Italy, as well as many surgeons, with only short‐term benefit.

**FIGURE 1:**
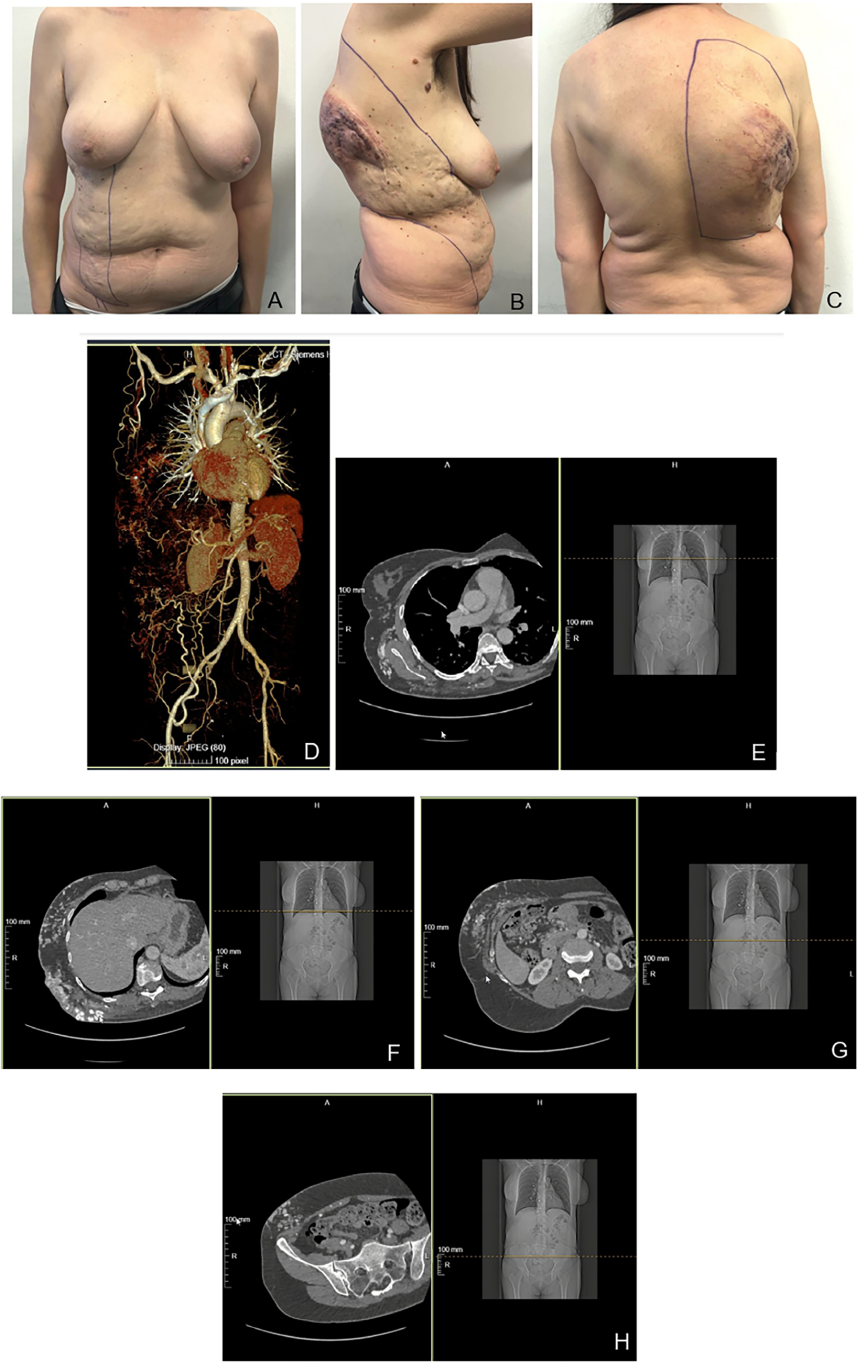
Pre‐operation picture of anterior (A), lateral (B), and posterior (C) views and pre‐operation AngioCT 3D reconstruction (D), multiple imaging of trunk (E and F), abdomen (G), and pelvis (H) AngioCT scan.

The mass kept growing, causing the patient physical and social discomfort and, most of all, chronic pain, being categorized as Schobinger stage III (Liu et al. 2010). When we first visited the patient, the mass was involving the right side of the body: hemipelvis, hemiabdomen, right side of her thorax, and hemi‐dorsum with estimated dimensions of 60 cm in length times 30 cm in width in the major points. It was warm, pulsatile, painful at rest, and gentle palpation with dystrophic skin, bluish discoloration, and some areas of ulceration (Figure 1A–C). The medical records of the patient include hypothyroidism and anxious‐depressive syndrome due to the life‐threatening condition and chronic pain. Two successful pregnancies and two cesarean deliveries were reported with no complications.

A multidisciplinary team has been organized, involving plastic surgeons, neurointerventional radiologists, oncologists, and thoracic surgeons. At first, an AngioCT study was performed to identify the extension of the mass, the vessels' origin of the AVM, and the involvement of the layers beneath it (Figure 1D–H). The radiological exam reported an involvement of the right anterior, lateral, and posterior thoracic wall and the right antero‐lateral abdominal wall, having as afferent vessels the right internal mammary artery, which was only partially embolized by the previous surgeries, the subscapular and lateral thoracic arteries, as well as the deep inferior epigastric artery and the deep circumflex iliac artery. According to Cho et al., angiographic classification is a Type IIIb (arteriovenous fistulae with dilated fistulae [Cho et al. 2006]). Our surgical plan consisted of an en bloc excision of the entire mass and the histopathological evaluation of the feeding vessels with an intraoperative biopsy examination.

After a multidisciplinary team discussion, our surgical plan consisted of a multistep approach to grant a more definitive and patient‐tailored treatment: first step—mass surgical extensive excision with possibly free margins; second step—selective angiography and eventually selective embolization of the remaining pathological vessels; third step—free flap reconstruction. The patient was accurately counseled, explaining the major possible risks such as unsuccessful eradication of the mass, massive bleeding, infection, flap necrosis, and even possible life‐threatening complications. The patient was aware of the possible risks, but chronic pain was an impediment for everyday living as well as the constant preoccupation of a possible life‐threatening rupture and bleeding.

**FIGURE 2:**
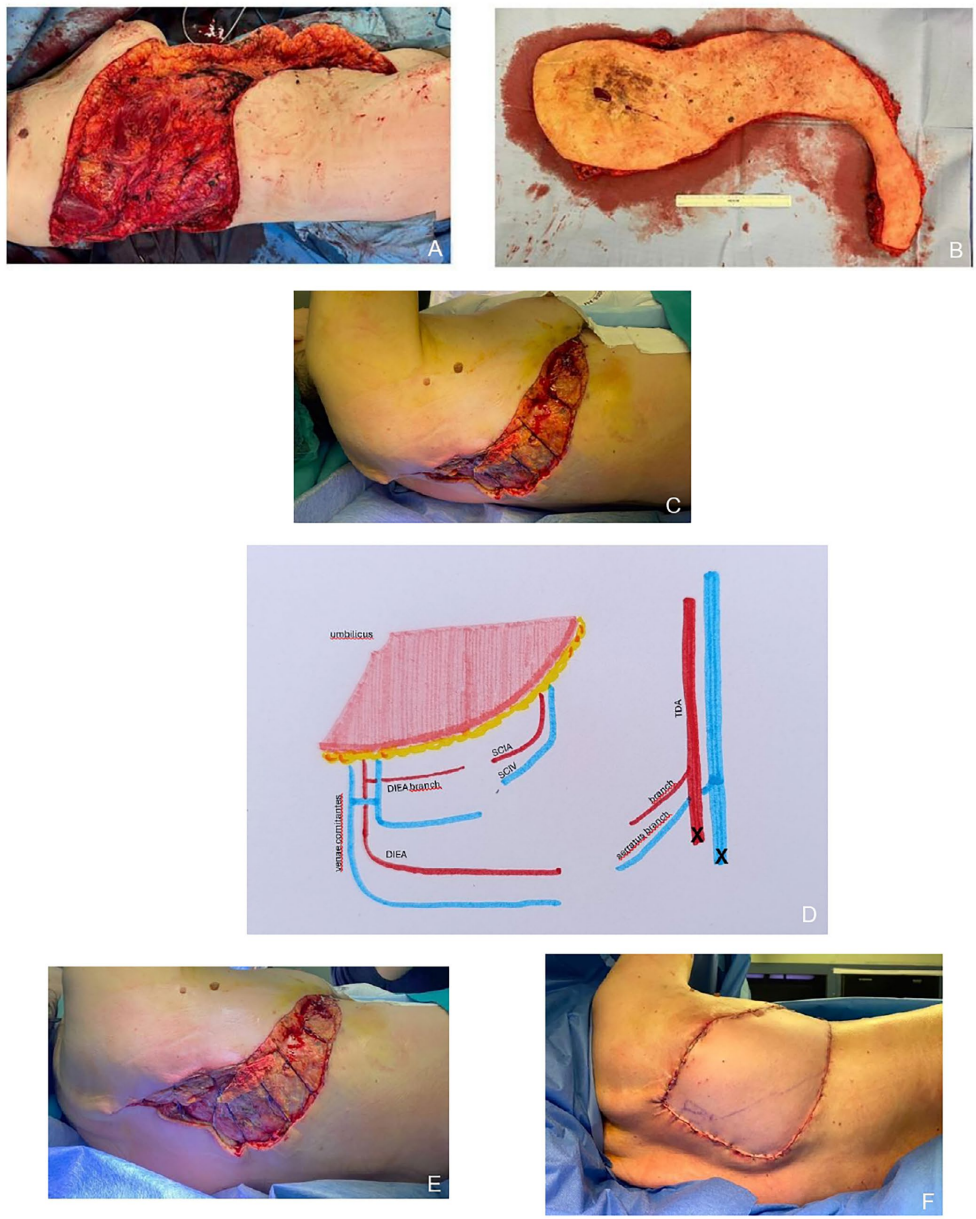
Intra‐operation images: AVM excision completed (A), excised AVM specimen (B), surgical gap from a lateral vision (C), schematic illustration of the performed anastomoses (D), surgical gap from a posterior vision (E), posterior surgical gap reconstructed with DIEP flap (F).

The surgical operating theater was prepared to have our intensivists on call and with a machine for surgical washed blood salvage technology and two red blood cells and two plasma units available in case of sudden hemorrhage. The patient was settled in a left lateral decubitus position, as we usually use for a latissimus dorsi raising flap position, but with a slight posterior torsion of the pelvis. At first our plastic surgery team performed an en bloc excision of the entire mass in a step by step excision, starting in the right pelvis, by following the lesion up until the right scapular and paravertebral region. An accurate hemostasis was needed, both to evaluate the condition of the tissue surrounding the AVM as also to avoid massive blood loss. The main afferent vessels have been identified and clamped, and intraoperative biopsies were sent: the thoracodorsal fragment still presented pathological dysplastic intima alterations, and a widened biopsy was performed. The AVM macroscopically involved the abdominal rectus right muscle, the right latissimus dorsi muscle, the anterior serratus muscle, and partially even the paravertebral muscles, which have been excised en bloc with the AVM (Figure 2A,B). During the first surgical step, small feeding vessels were spared and marked with clamps for the neurointerventional radiologist to define AVMs entrance point for possible embolization procedure. The anterior surgical gap has been easily closed by primary intention. The dorsal surgical wound was too wide, so we could only temporarily approximate the wedges (Figure 2C,E) and use an iodoform dressing, sterile gauzes, and a sterile coverage dressing. Two drainages, subfascial and subcutaneous, were positioned.

The second surgical step has been scheduled after 48 h of intensive care postoperation observation. In this period of time the patient was monitored and kept under deep sedation by our intensivists. The neurointerventional radiologists performed an embolization of a paravertebral arteriovenous hypertrophic shunt at T7 level, which was the only pathological evidence that remained.

After four more days of postoperation observation (i.e., 6 days after the first surgery), the third surgical step was performed. The best reconstructive option considering its width and thickness appeared to us to be the DIEP flap. The perforators of the left deep inferior epigastric artery have been identified from the pre‐operation AngioCT scan and did not present any pathologic alteration; the day before surgery, they were also identified and marked on the patient's skin using an ultrasound Doppler exam.

For the last surgical step, the patient was positioned, at first, in a supine position. The left superficial inferior epigastric vein and artery were identified and carefully isolated for an adequate length of 8 cm. The flap was raised medial‐laterally since we already had a wound on the right hemiabdomen. Two perforators were identified; the incision of the deep fascia performed for the complete AVM excision on the right abdomen was used to complete the subfascial dissection of the perforators up to its origin. We also identified the superficial circumflex iliac artery and vein, which had been accurately dissected until we reached an adequate diameter and length. In order to assure flap viability, we performed an intraoperative ICG test, which proved a good perfusion from both vascular axes before raising the flap. A primary closure with no tension of the donor site was performed, and the neoumbilical plasty was tailored. We positioned one drainage per side. On a separate sterile table, we anastomosed the pedicle of the superficial circumflex iliac artery and vein with one collateral artery of the DIEA and its comitante vein.

The patient was positioned in a left lateral decubitus for insetting and microsurgical anastomosis. The wound dressing and previous approximation sutures were removed, leaving a surgical gap of 30 × 10 cm. Another intraoperative biopsy of the thoracodorsal artery has been sent to assure its healthy condition. The venous microanastomosis was performed between a venous branch of the right serratus muscle with 8/0 single stitches since the thoracodorsal vein appeared to be too fragile during its preparation. After the result of the biopsy exam, the arterial microanastomosis was made between a collateral branch of the thoracodorsal artery and the DIE artery (Figure 2D). Positive patency test, good capillary refill test, and a good bleeding flap margin reassured us of the flap's optimal perfusion. We also performed another ICG test to control its perfusion and viability. After positioning a dorsal drainage, the wound was closed (Figure 2F), and medications were placed.

The patient received 4000 IU of heparin 12 h after surgery and continued her antibiotic prophylactic treatment. Drainages were removed on the fourth postoperatory day and on the ninth, the patient was discharged from the hospital without complications. The patient immediately referred a diminished pain sensation. The patient was followed up in our outpatient clinic at 1 week, 2 weeks, 1 month, and 3 months and repeated an Angio‐MRI with our interventional radiologists at 6‐month follow‐up.

**FIGURE 3:**
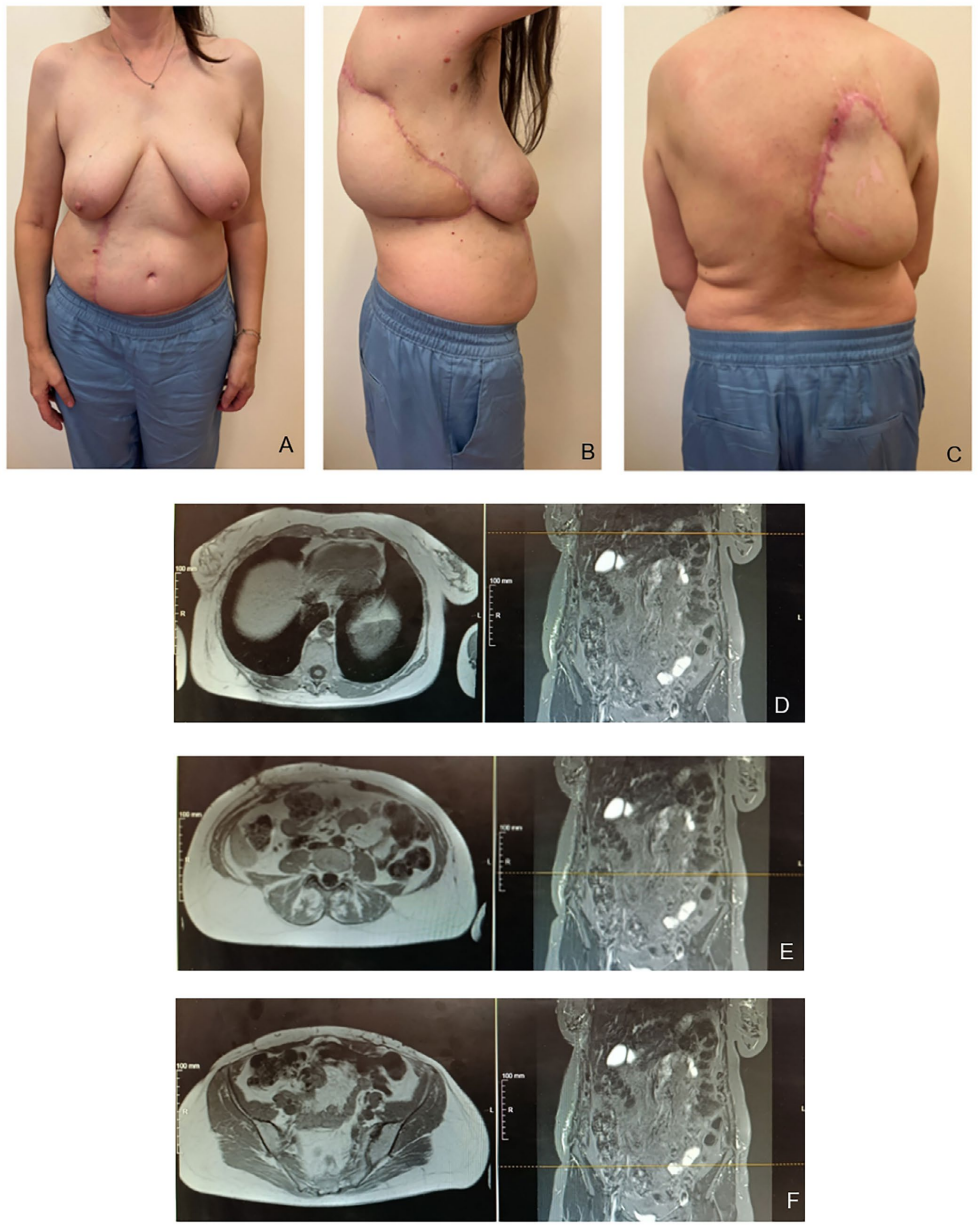
Postoperation pictures at 6 months, respectively, anterior (A), lateral (B), and posterior (C), and iodine contrast‐MRI multiple imaging of trunk (D), abdomen (E), and pelvis (F).

The patient was hospitalized for 15 days, the first two postop days in sub‐intensive care unit for close monitoring, and afterward transferred to our department. During hospitalization, the patient started adequate physiotherapy to gain proper function in the right arm. After proper functional gain and strength, pain control, and achieving normal blood exams, the patient has been dismissed and started a close follow‐up during the first month, then at 3 months and at 6 months. At 6 months, the patient described the result as “good” with benefit during daily life activities, pain control, and quite a psychological benefit from surgery (Figure 3A–C). We performed an MRI with contrast in order to check if there were any residues of AVMs (Figure 3D–F). The test reported no significant signs of pathological residues.

**FIGURE 4:**
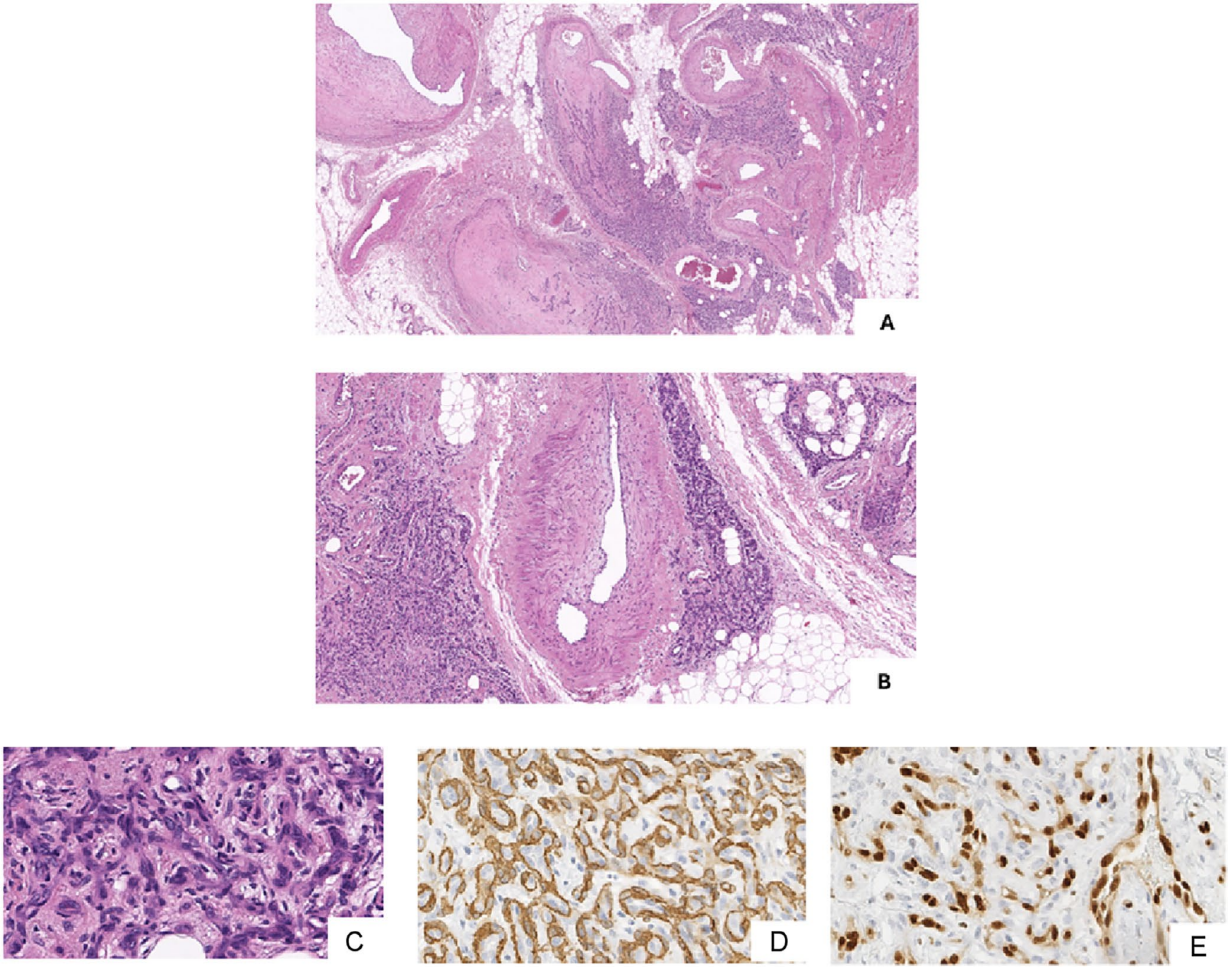
Multiple ample areas of microvascular proliferation encircling the large vessels of an arteriovenous malformation (A, H&E, 2×; B, H&E, 5×) and proliferation of small vessels with swollen endothelia and inconspicuous lumina (C, H&E, 40×); continuous pericyte layers highlighted by actin‐1‐A4 immunostain (D, H&E, 40×), endothelial cells displaying nuclear enlargement (E, ERG immunostaining, 40×).

Morphological examination of the surgical specimen was consistent with deep AVM, showing large numbers of tortuous arteries with fibrointimal thickening. Multifocal areas of microvascular proliferation were seen in between the otherwise mature vessels of the malformation (Figure 4).

## 3 Discussion

AVMs are a rare condition caused by an error during embryogenesis that leads to an abnormal communication between arteries and veins, such as a nidus or fistulas, bypassing the complex capillary system. This high‐flow shunt draws blood from the system and, as a consequence, is more likely to rupture due to its dilated and fragile walls (Young and Mulliken 1988). AVMs are 20 times more common in the central nervous system because apoptosis here is rare (Gomes and Bernatz 1970). The extra‐cerebral AVMs are commonly localized in the neck and head, more rarely in the limbs. The development of AVMs in the trunk is extremely rare, so this condition is sporadically cited in literature (Spiotta et al. 2011; McCarthy et al. 2018; Parashi et al. 2013; Houbballah et al. 2010; Ashley et al. 2006; Yilmaz et al. 2010). The management of AVMs depends on their Schobinger staging: from conservative observation to medical treatment, percutaneous embolization, surgical eradication, and also palliative surgery (Greene and Orbach 2011; Visser et al. 2011; Widlus et al. 1988; Houbballah et al. 2010; Vaišnytė et al. 2012; Liu et al. 2010). The main goal of treatment is blocking the disease's natural history, which leads to a slow but continuous enlargement, with higher risk of rupture so therefore bleeding, functional impairment, social discomfort, and even life‐threatening conditions due to heart failure consequences (Liu et al. 2010). This condition's treatment should always be carefully planned by a multidisciplinary team. It is usually treated in steps, involving a percutaneous conservative therapy that can allow for a more safe surgical eradication (Spiotta et al. 2011; Chun et al. 2015; Zobel et al. 2021; Widlus et al. 1988; Yilmaz et al. 2010). In our case, multiple attempts at endovascular treatments have been completed with no long‐lasting benefits. On the contrary, it created a major blood inflow in the remaining pathological vessels, which caused a massive enlargement of the persisting lesion. The previous partial and non‐radical resections were responsible for a self‐maintenance enlarging mechanism. In fact, it is described that a non‐radical excision lead to an augment of the mass and of the risks connected with this phenomenon; that is why it is so important to perform en bloc surgical excision (Zobel et al. 2021; Liu et al. 2010; Cho et al. 2006). The role of the anatomopathologist was crucial to prove radicality before reconstruction. The histopathological evaluation of the feeding vessels with intraoperative and definitive biopsy examination for dysplastic intima alterations helped us to define a healthy vascular territory. Our decision to perform an anastomosis on a separate sterile table was to allow the head surgeon to have more space and immobility while the surgical team could continue performing accurate hemostasis and closure, thus decreasing the surgical timing. We performed an en bloc surgical excision followed by a procedural arteriography to control possible remaining pathological vessels that were closed by embolization. We discussed with the neurointerventional surgeons to wait 48 h after the en bloc excision before performing the embolization in order to allow for an eventual opening of collateral pathological vessels that may require our attention. The en bloc excision caused a massive soft tissue loss that required reconstruction with a free flap.

Areas of microvascular proliferation occur in up to 30% of congenital vascular malformations, especially those with high flow characteristics (Meijer‐Jorna et al. 2013). It has been hypothesized that these vascular growths may represent an angiogenic response triggered by hypoxia and/or inflammatory cytokines (Meijer‐Jorna et al. 2012).

The onset of microvascular proliferation appears to be unrelated to sex, age, or location of AVMs (Meijer‐Jorna et al. 2007). However, when present in congenital vascular malformations of skin and soft tissue, it may result in symptomatic growth in a subpopulation of patients (Meijer‐Jorna et al. 2013).

Microvascular proliferations consist of closely packed small vessels with narrowed lumina and swollen endothelia and raise suspicion for angiosarcoma, which has been reported, though very rarely, in association with AVMs. Improving awareness of these modifications among pathologists would help to avoid overdiagnosis of malignancy.

In literature, there are few cases of plastic reconstructive surgery following AVMs surgical treatment (Kohout et al. 1998; Visser et al. 2011). AngioCT scan can provide surgeons with clear identification of the feeding vessels, organizing a more accurate surgical plan as well as studying the possible perforators needed for a microsurgical reconstruction. In literature there are few cases reconstructed with a free flap: groin, ulnar forearm, deep circumflex iliac artery, latissimus dorsi, gracilis, and rectus abdominis free flaps were used and described (Visser et al. 2011; Spiotta et al. 2011; Zobel et al. 2021). We planned a DIEP free flap since it would have as little impact as possible on the patient's functional abilities by sparing muscles, since it could provide large coverage, and since it would cause a less visible scar stigma. We believe that a longer follow‐up will benefit the patient to control an eventual recurrence of the AVM and to check if the patient has any correction desire. The principal limitation of our surgical approach is the patient's health condition, morbidity, and determination. We could not perform this three‐step surgical approach if the patient wasn't in optimal health condition. We could not perform this three‐step surgical approach if the patient was not in optimal health conditions. It is also fundamental to have a determined patient in order to perform physiotherapy as soon as possible. We are strongly convinced that a quick mobilization increases the patient's well‐being, and it is essential in order to decrease possible complications such as urinary tract infections, pressure wounds, respiratory distress, and an aggravation of her anxious‐depressive syndrome. This multiple‐step surgery could offer future patients a curative surgical treatment with less social impairment and accelerated full recovery in these complex massive cases.

## Author Contributions

G.M. performed the surgery with I.E.M., S.T., and A.L.A. R.S. analyzed and examined the histological samples, as well as edited and refined the anatomopathological section of the manuscript. I.E.M. wrote the manuscript with support S.T. and A.L.A. G.M., F.M., and I.A. helped supervise the project and the final revision.

## Funding

The authors have nothing to report.

## Conflicts of Interest

The authors declare no conflicts of interest.

## Data Availability

The data that support the findings of this study are available on request from the corresponding author. The data are not publicly available due to privacy or ethical restrictions.
